# Leukemic phase and CSF involvement of diffuse large B‐cell lymphoma with a complex karyotype including a *TP53* deletion

**DOI:** 10.1002/ccr3.1250

**Published:** 2017-12-05

**Authors:** Jillian Sinkoff, Horatiu Olteanu, Jess F. Peterson, Nirav N. Shah

**Affiliations:** ^1^ Department of Medicine Medical College of Wisconsin Milwaukee Wisconsin; ^2^ Department of Pathology Froedtert/MCW Laboratory Building Milwaukee Wisconsin

**Keywords:** DLBCL, NHL, TP53 deletion

## Abstract

Diffuse large B‐cell lymphoma in rare instances can present initially in a leukemic phase and mimic other lymphoid diseases. In such cases, advanced diagnostic testing including immunophenotyping, FISH analysis, and karyotyping can help determine the accurate diagnosis which is key in the management of the disease.

## Introduction

A 59‐year‐old presented to oncology clinic with a 15‐lb. weight loss, right‐sided Bell's palsy, and back pain. Complete blood count (CBC) at consultation revealed a WBC count of 130,800/*μ*L with 72% unclassified cells, hemoglobin of 6.4 g/dL, and platelets of 27,000/*μ*L. Additional laboratory work demonstrated an LDH of 8672 units/L, uric acid of 10.2 mg/dL, and potassium of 5.3 mmol/L. Two weeks' prior the patient's CBC demonstrated a WBC count of 7600/*μ*l, hemoglobin of 9.5 g/dL, and platelets of 112,000/*μ*L.

**Figure 1:**
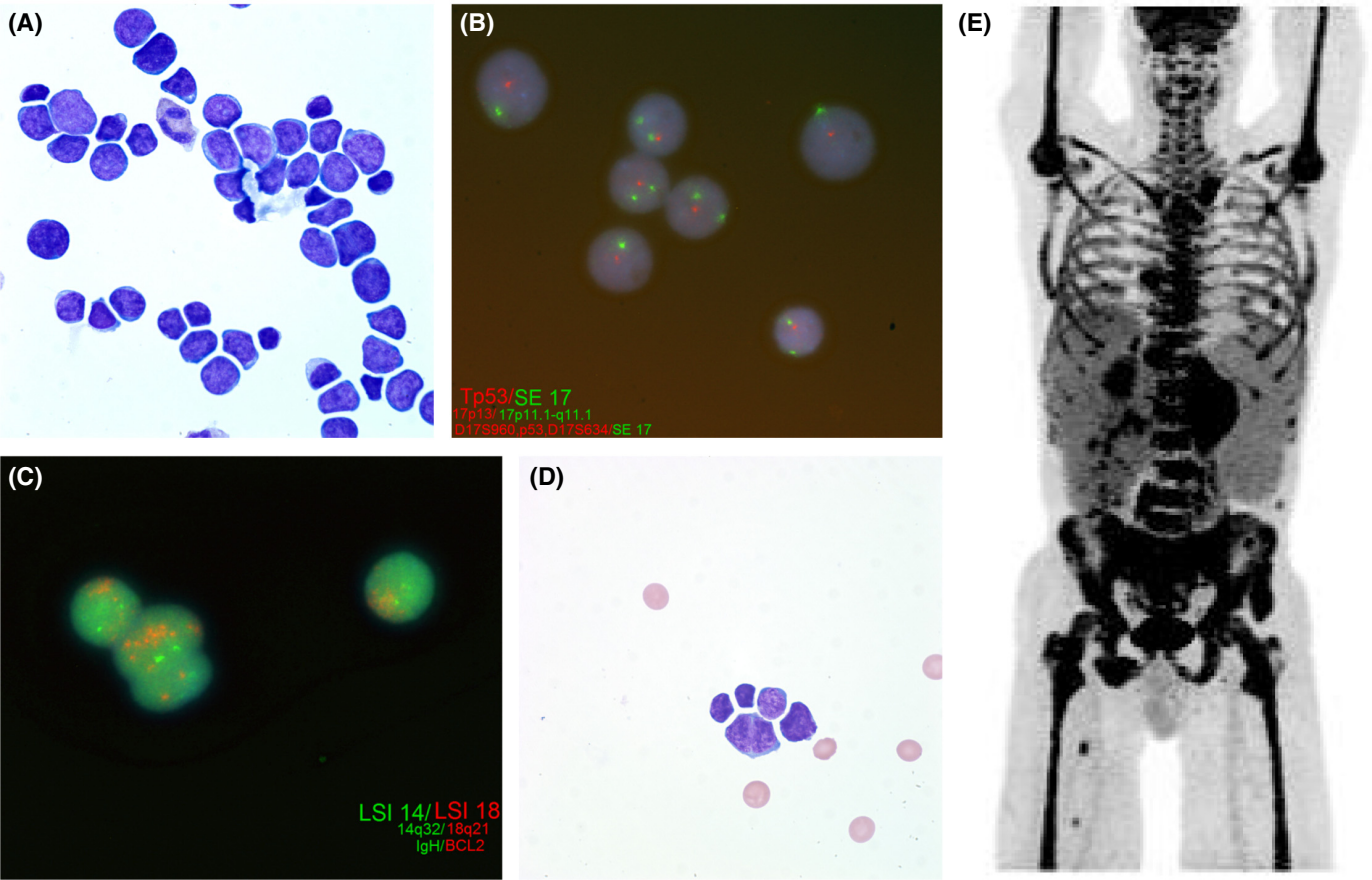
(A) Peripheral blood smear demonstrating leukemic involvement with DLBCL (B) FISH study demonstrating *TP53*/17p deletion in 83.5‐90% of involved cells (C) BCL2 amplification (red signals) (D) CSF involvement with DLBCL (E) PET/CT demonstrating widespread marrow and extranodal involvement.

A peripheral smear demonstrated a predominant population of abnormal medium‐ to large‐sized lymphoid cells with mildly irregular to clefted nuclei, coarsely dispersed chromatin, prominent nucleoli, and small to moderately abundant basophilic cytoplasm (Fig. 1A). An additional subset of small lymphocytes with high nuclear to cytoplasmic ratio, regular nuclei, variably condensed chromatin, and small nucleoli, was also noted. Peripheral blood flow cytometry showed two distinct B‐cell clones. The main population (69% of events) consisted of medium‐ to large‐sized B cells that were CD10+, CD19 variably dim+, CD20+, CD22 variably dim+, CD38 bright+, CD45+, FMC‐7+, kappa+, CD5‐, CD23‐, and lambda‐. The minor population (11%) consisted of small‐sized cells that were CD19 variably+, CD20+, CD22, variably dim+, CD5 partial+, CD10 partial+, FMC‐7 bright+, CD45+, kappa+, CD38‐, CD23‐, and lambda‐. Fluorescence in situ hybridization (FISH) was negative for gene rearrangements of *BCL2*,* BCL6*,* CCND1*, and *MYC*, but was positive for trisomies 3, 8, and 12, distal 13q duplication, *TP53* deletion (Fig. 1B), and amplification of *BCL2* (Fig. 1C). Peripheral blood karyotype demonstrated complex karyotype with multiple consistent structural and numerical abnormalities. While FISH results and CD5 positivity may be suggestive of a Richter's (large cell) transformation or chronic lymphocytic leukemia/small lymphocytic lymphoma, the overall morphologic and immunophenotypic findings, along with amplification of *BCL2,* were felt to be most consistent with diffuse large B‐cell lymphoma (DLBCL). A diagnosis of leukemic phase large B‐cell lymphoma was made. CSF analysis demonstrated involvement with a CD5 subset+, CD10+, CD19+, CD20+, CD38+, kappa light‐chain‐restricted B‐cell population (Fig. 1D). PET/CT demonstrated liver lesions, large paraspinal mass, osseous involvement, and soft tissue/muscle deposits consistent with lymphomatous involvement (Fig. 1E).

Leukemic presentation of DLBCL is a rare entity that often presents with significant extranodal involvement.^1^, ^2^ This patient with *TP53* deletion and complex cytogenetics received R‐Hyper‐CVAD chemotherapy with twice‐weekly intrathecal (IT) therapy (alternating methotrexate and cytarabine) for two cycles. His induction was complicated by tumor lysis syndrome and infection. Due to refractory CSF involvement, his therapy was modified to R‐ICE, and IT thiotepa was added to his regimen. He ultimately cleared his CSF after >2 months of systemic and IT chemotherapy. He then received consolidative craniospinal irradiation prior to proceeding with an allogeneic transplant in first remission. Unfortunately, despite aggressive measures, he experienced CNS relapse at day 50 postallogeneic transplant.

## Authorship

JS and NS: contributed to the writing, drafting, and editing of the manuscript; approved the final version of the manuscript; and acquired case data. HO: contributed to the writing, drafting, and editing of the manuscript; approved the final version of the manuscript; and acquired pathology images used in the manuscript. JFP: contributed to the drafting and editing of the manuscript; approved the final version of the manuscript; performed FISH with interpretation; and acquired images used in the manuscript.

## Conflict of Interest

The authors declare that they have no competing interests.
